# Supplementary material for: Cameroonian Spice Extracts Modulate Molecular Mechanisms Relevant to Cardiometabolic Diseases in SW 872 Human Liposarcoma Cells
Source: Nutrients. 2021 Nov 26;13(12):4271. doi: 10.3390/nu13124271 (PMC8706885; doi:10.3390/nu13124271)
Supplement: Supplementary file 1 [file nutrients-13-04271-s001.zip › nutrients-1479355-supplementary.pdf]

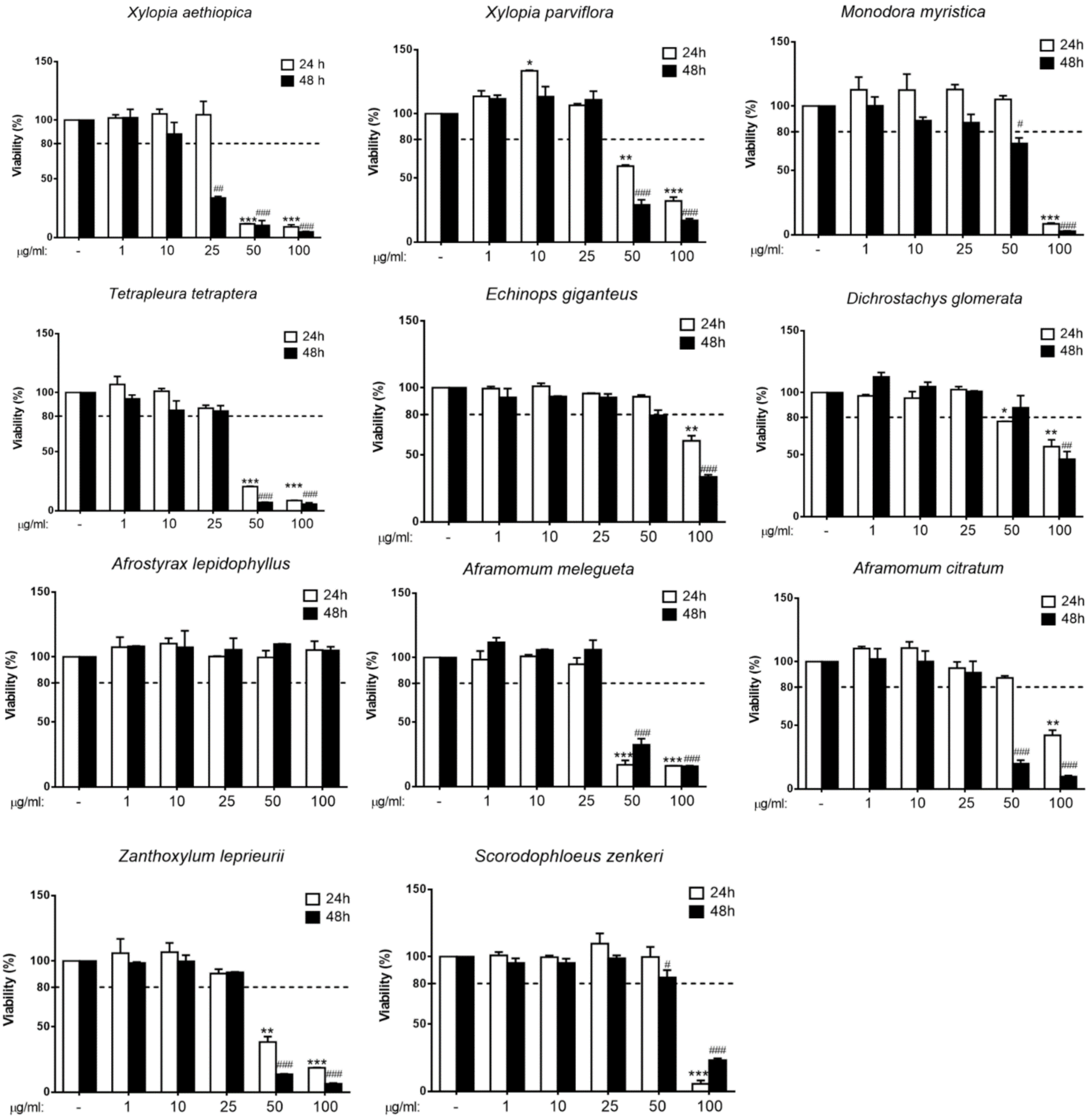

Supplementary Figure S1. SW 872 cells viability (MTS assay) after treatment with all extracts. Cells were incubated for 24 h and 48 h with increasing concentrations (1 - 100 μg/mL) of each extract. Data are expressed as % of control taken as 100; mean±SD, N=3. The dotted line set at 80% indicates the threshold above which cells are considered viable. * $p < 0.05$, ** $p < 0.01$, *** $p < 0.001$ vs. the control group of 24 h incubation; # $p < 0.05$, ## $p < 0.01$ and ### $p < 0.001$ vs. the control group of 48 h of incubation.

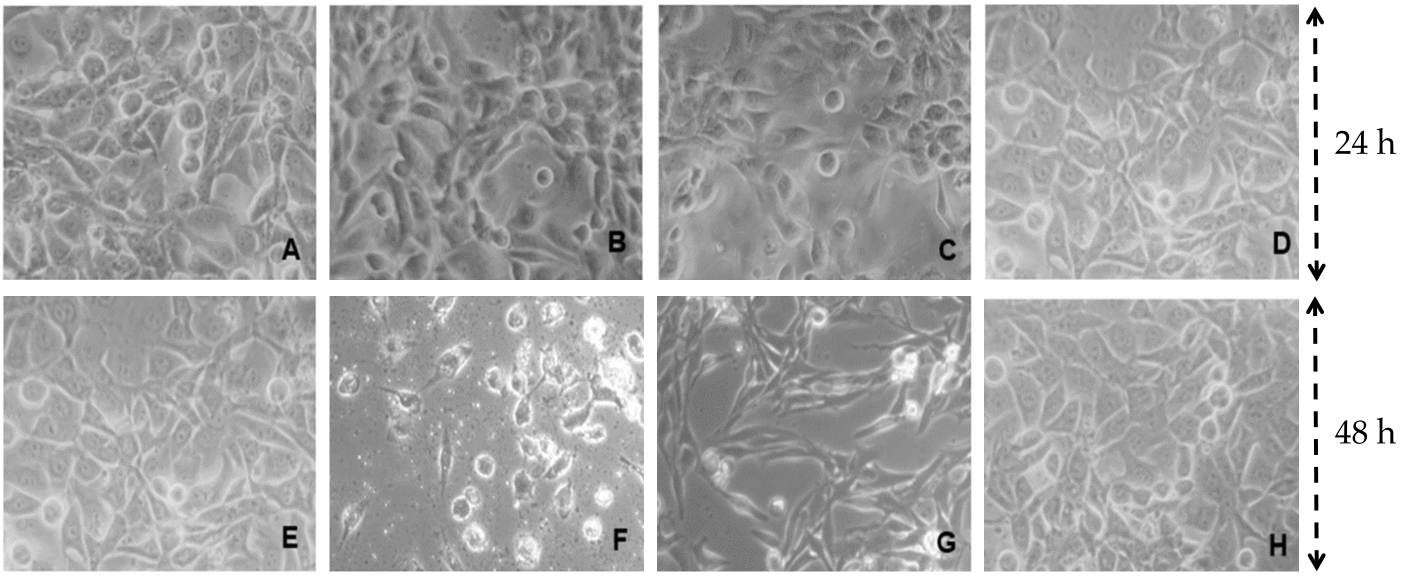

Supplementary Figure S2. Morphological analysis of differentiated SW 872 cells treated with spice extracts. (A) control cells, incubation: 24 h; (B) treatment with 10 µg/mL *Xylopia aetiopica,* 24 h; (C) treatment with 20 µg/mL *Xylopia aetiopica,* 24 h; (D) treatment with 10 µg/mL *Afrostyrax lepidophyllus,* 24 h; (E) control cells, incubation: 48 h; (F) treatment with 100 µg/mL *Xylopia aetiopica,* 48 h; (G) treatment with 100 µg/mL *Afrostyrax lepidophyllus,* 48 h; (H) treatment with 20 µg/mL *Afrostyrax lepidophyllus,* 24 h. Images were obtained using a light microscope at 20 X magnification.

Effect of spice extracts on triglyceride accumulation in differentiated SW 872 cells.

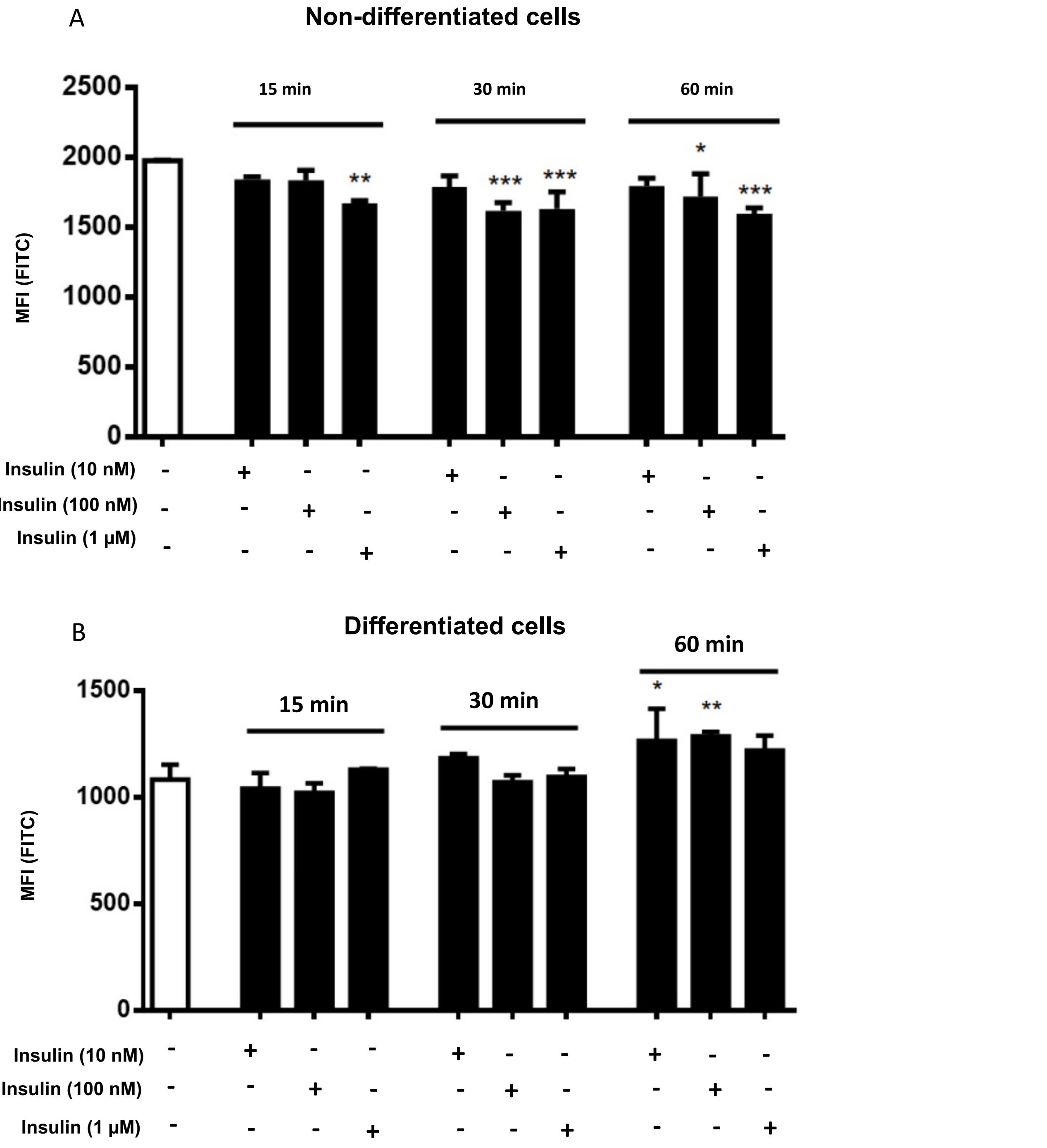

Supplementary Figure S3. Glucose uptake in SW 872 cells. Time-course (15-60 min) and dose-response to insulin (10 nM-1 µM) of glucose uptake in non-differentiated (A) and differentiated (B) cells. Glucose uptake was assessed by FACS analysis. One experiment (n=3) is shown, representative of 3 separated experiments, each in triplicate. Results are expressed as mean±SD. * $p < 0.05$, **$p < 0.01$, *** $p < 0.001$ (one-way ANOVA multiple comparison).
